# Susceptibility- and T2*-weighted MRI features of CNS large B-cell lymphoma in a large single-center cohort: prevalence, patterns, and clinical associations

**DOI:** 10.1007/s11060-025-05124-8

**Published:** 2025-07-12

**Authors:** Christophe T. Arendt, Marie Löhlau, Linda Röder, Michael C. Burger, Elke Hattingen, Stefan Weidauer

**Affiliations:** 1 Institute of Neuroradiology, Goethe University Frankfurt, University Hospital, Frankfurt am Main, Germany; 2 Dr. Senckenberg Institute of Neurooncology, Goethe University Frankfurt, University Hospital, Frankfurt am Main, Germany

**Keywords:** Cerebrum, Diffuse Large B-cell lymphoma, Magnetic resonance imaging, Neuroimaging, Primary central nervous system neoplasms

## Abstract

**Purpose:**

The prevalence of susceptibility effects (SE) on T2*-weighted imaging (WI) and susceptibility-WI (SWI) in primary large B-cell lymphoma (IP-LBCL) of the central nervous system (CNS) and diffuse LBCL (DLBCL) with secondary CNS lymphoma (SCNSL) remains debated. This study aimed to clarify SE prevalence and their associations with primary versus secondary manifestations, immune status, corticosteroid treatment, and structural MRI features.

**Methods:**

This retrospective, single-center study included histologically confirmed DLBCL cases (WHO ICD-10 C83.3) with intracerebral involvement (March 2011–November 2023). Subjects without cranial MRI or diagnostic susceptibility-based sequences were excluded. T2*WI and SWI were independently reviewed by two neuroradiologists for presence or absence of SE. Identified SE were classified into five types: punctate, linear, confluent, conglomerate, and/or ring-like. Lesion focality and morphology were assessed on T2WI and contrast-enhanced T1WI. Clinical data included extracranial lymphoma history, immune status, and corticosteroid initiation.

**Results:**

Among 128 cases (median age: 70 years [58–75]; 65 men), 119 (93%) had IP-LBCL and 9 (7%) had SCNSL. 110 (85.9%) subjects were immunocompetent. T2*WI was available in 90 (70.3%) datasets and SWI in 38 (29.7%). SE detection was higher on SWI (71.1%) than T2*WI (47.8%; *P* = 0.03). No association was found between SE and lymphoma type (*P* = 1.00). In IP-LBCL, immunosuppression was significantly associated with SE presence (*P* = 0.001; OR = 16.1, 95% CI: 2.89–304.79), while age, gender, and corticosteroid use (18.5%) were not. SE showed no significant associations with structural imaging features, including necrosis.

**Conclusion:**

SE are common in both IP-LBCL and SCNSL, particularly on SWI, and present with variable patterns unrelated to structural MRI features. In IP-LBCL, immunosuppression, but not pre-existing corticosteroid treatment, is significantly associated with the presence of SE.

**Supplementary Information:**

The online version contains supplementary material available at 10.1007/s11060-025-05124-8.

## Introduction

In adults, lymphomas rank as the second most common type of primary malignant central nervous system (CNS) tumors after gliomas [1–3], with knowledge of neuroradiological features being crucial for differential diagnosis, particularly to avoid corticosteroid administration prior to performing a biopsy [2, 4–7]. The most prevalent subgroup, by far, is primary diffuse large B-cell lymphoma (DLBCL) of the CNS [1–4]. Per the 5th edition of the World Health Organization classification of hematolymphoid tumors/lymphoid neoplasms, these are categorized as primary large B-cell lymphomas of immune-privileged sites (IP-LBCL) [3, 8]. In cases of immunodeficiency or immune dysregulation, lymphomas linked to Epstein-Barr virus are termed immunodeficiency-associated CNS lymphomas [1, 3, 8]. Another, rarer form is secondary CNS lymphoma (SCNSL), which can emerge from prior extracerebral manifestations of DLBCL, presenting either at initial diagnosis (synchronous) or as a subsequent relapse (metachronous) [2, 5].

The neuroradiological characteristics of IP-LBCL, defined through traditional and advanced imaging over the past two decades [9–17], have shown variability, especially concerning susceptibility effects (SE) on T2*-weighted imaging (WI) and susceptibility-WI (SWI) [2, 17]. Those SE on MRI reflect various underlying pathophysiological processes, such as presence of hemosiderin from hemorrhages, iron-laden microglia or macrophages, and calcifications, which would require histopathological analysis for definitive differentiation. Earlier studies considered the absence or rarity of SE typical for IP-LBCL, differentiating it from malignant gliomas [18–28]. However, subsequent studies indicate that SE may appear in up to 50% of cases [2, 17, 29]. Hemorrhage, along with necrosis, is a characteristic finding in immunodeficiency-associated lymphomas  [2, 4]. Advances in the treatment of human immunodeficiency virus infections have shifted the focus toward autoimmune-associated immunocompromise, post-transplantation conditions, and immunomodulatory therapies [2, 30–32]. DLBCL with secondary CNS lymphoma (SCNSL) are less well characterized, typically showing parenchymal involvement as compared to leptomeningeal involvement seen in SCNSL from Burkitt or mantle cell lymphoma [2, 5]. This study aimed to elucidate the prevalence of SE in a large DLBCL cohort and its associations with primary and secondary CNS involvement, immune states, the effects of previously administered corticosteroid doses, and structural MRI features.

## Methods

### Study design, patient cohort and baseline characteristics

This retrospective, observational study was conducted as a single-center investigation, which was approved by the Ethics Committee of the Faculty of Medicine of the Goethe University Frankfurt, Germany (reference: 2023-1367) and conducted in accordance with the principles of the Declaration of Helsinki. The recruitment list was obtained from the local University Center for Tumor Diseases database (project number: UCT-4-2023). It included histologically confirmed DLBCL cases classified under code C83.3 of the World Health Organization (WHO) International Classification of Diseases, Tenth Revision, with primary or secondary cranial involvement identified through institutional codes corresponding to the head region. Cases were consecutively collected from March 2011 to the retrieval date in November 2023 from the University Center for Tumor Diseases. Exclusion criteria were applied for cases without cranial MRI in the internal Picture Archiving and Communication System (e.g., external images not uploaded, only cranial CT or whole-body PET-CT available, or MRI contraindicated), cranial MRI lacking T2*WI or SWI sequences, technically inadequate susceptibility-based images due to artifacts (e.g., severe motion artifacts, pronounced susceptibility artifacts caused by implants), or absence of intracerebral manifestations on cranial MRI (i.e., extracerebral findings only). Baseline characteristics for all subjects, including demographic, clinical, and diagnostic data, were obtained from the local clinical information system through manual data abstraction from medical reports at the time of the initial CNS lymphoma diagnosis.

### Cranial magnetic resonance imaging

In cases of IP-LBCL, the scan nearest to the brain biopsy that met the criteria was analyzed, while for DLBCL with SCNSL, the earliest image dataset demonstrating CNS involvement was selected. Susceptibility-based MRI sequences acquired within the institution were performed using the 1.5 Tesla scanner ((1) Achieva dStream, Philips, Netherlands) and the 3.0 Tesla scanners ((2) MAGNETOM Verio and (3) MAGNETOM Skyra, Siemens Healthineers, Germany), with the following technical parameters: for T2*WI, (1) repetition time: 519 ms, echo time: 14 ms, flip angle: 18˚, in-plane spatial resolution: 0.7 × 0.7 mm, slice thickness: 5.0 mm, acquisition matrix: 296 × 236, reconstruction matrix: 320 × 320; (2) and (3) 631 ms, 20 ms, 20˚, 0.4 × 0.4 mm, 5.0 mm, 256 × 216, 432 × 512; and for SWI (1) 52 ms, 0 ms, 20˚, 0.3 × 0.3 mm, 2.5 mm, 260 × 255, 672 × 672; (2) 27 ms, 20 ms, 15˚, 0.9 × 0.9 mm, 1.5 mm, 192 × 256, 256 × 182; (3) 27 ms, 20 ms, 15˚, 0.9 × 0.9 mm, 1.5 mm, 256 × 223, 232 × 256. For external datasets, the slice thicknesses were in a range of 5.0 to 6.0 mm for T2*WI and of 1.0 to 3.0 mm for SWI. Phase-sensitive images from SWI were not consistently available across datasets, particularly in external scans, and were therefore not included in the analysis. All scans were independently reviewed by two experienced neuroradiologists to identify regions of SE within the tumors. These regions were defined as areas with markedly reduced signal intensity compared to normal-appearing brain parenchyma. Any discrepancies between the reviewers were resolved through a consensus reading conducted later to minimize recall bias. Following consensus, one of the reviewers categorized the patterns of signal changes on SWI or T2*WI into five distinct types based on their visual appearance: punctate (small, isolated, dot-like regions), linear (small, isolated, line-like regions), confluent (merged punctate and/or linear areas forming continuous regions), conglomerate (dense clusters), and ring-like (circular or partially circular regions surrounding a central area) (Fig. S5). In addition to evaluating SE, the morphological patterns were analyzed using T2WI and contrast-enhanced T1WI (ce-T1WI). This analysis included lesions that were solid and enhancing, without or with necrosis; necrotic and ring-like enhancing; diffuse parenchymal that were either enhancing or non-/scarcely enhancing (lymphomatosis cerebri-like); diffuse perivascular and enhancing; diffuse cortical/pial and enhancing; or diffuse ependymal and enhancing. Moreover, the presence of uni- or multifocality was determined, with the latter further categorized based on whether the patterns observed on T2*WI/SWI and T2WI/ce-T1WI were consistent or varied between the tumors. Multiple entries were possible for cases with differing imaging characteristics across sequences.

### Statistical analysis

All statistical analyses were performed using R software (version 4.3.0). The significance level α was set at 5%, with *P*-values < 0.05 considered statistically significant. The normality of continuous data was assessed using the Shapiro–Wilk test for smaller sample sizes and the Lilliefors-corrected Kolmogorov–Smirnov test for larger datasets. Continuous variables were summarized as means with one standard deviation or medians with interquartile ranges [first quartile—third quartile], depending on their distribution. To compare continuous variables between groups, Mann–Whitney *U* tests were used for nonnormally distributed data. Categorical variables were reported as absolute frequencies, percentages, and proportions. For proportions, two-sample tests for equality of proportions with continuity correction were employed. Categorical variables were further compared using Pearson’s Chi-squared tests with Yates' continuity correction for 2 × 2 contingency tables or Fisher’s exact tests when cell counts were small. Odds ratios (OR) and their 95% confidence intervals were calculated to assess associations between categorical predictors and outcomes. The association between susceptibility patterns on T2*WI/SWI and structural features on T2WI/ce-T1WI was evaluated using Fisher's exact test, with a simulated *P*-value based on 10,000 Monte Carlo replicates due to the size and sparsity of the contingency table. Logistic regression analyses were performed to identify predictors of SE. Independent variables included age, gender, immunosuppressive status, and started corticosteroid treatment. Corticosteroid treatment status was categorized as “yes,” “no,” or “unknown.” The primary logistic regression model included all cases, with “unknown” treated as a separate category. A sensitivity analysis was then performed by excluding cases with unknown corticosteroid status to assess the robustness of the findings. Model fit was assessed using the Akaike Information Criterion and changes in deviance. Agreements between the two raters on the detection of intratumoral SE were evaluated using Cohen’s Kappa (κ) for nominal data. The strength of agreement was categorized as follows: κ = 0, poor; κ = 0.01–0.20, slight; κ = 0.21–0.40, fair; κ = 0.41–0.60, moderate; κ = 0.61–0.80, substantial; κ = 0.81–1.00, almost perfect.

## Results

### Baseline analyses

**Fig. 1:**
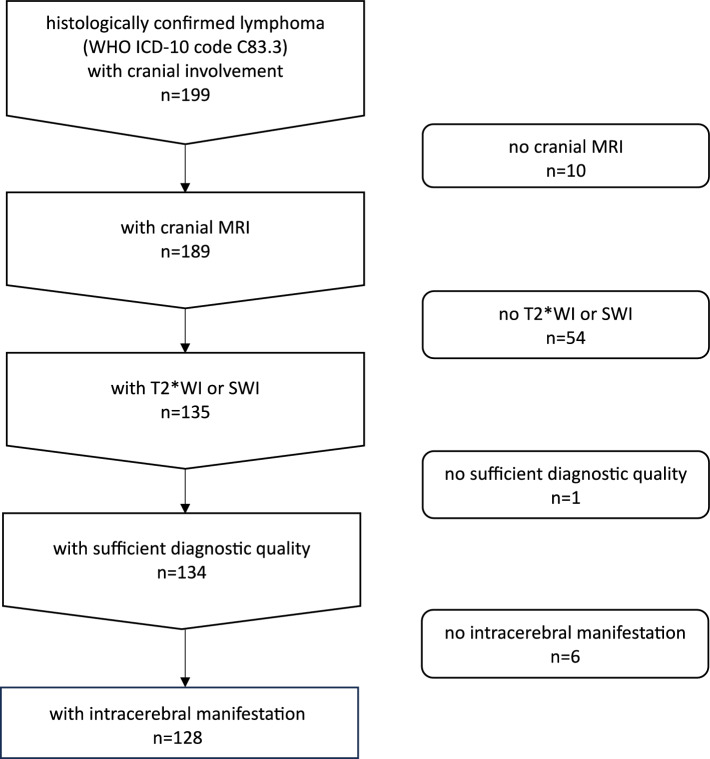
Flowchart of the study population depicting enrollment and exclusions

The recruitment process identified a total of 199 unique patients after removal of duplicate entries. Figure 1 provides a flow diagram summarizing the selection process, resulting in a final cohort of 128 subjects diagnosed with DLBCL with CNS involvement. This cohort had a median age of 70 years [58–75.25] (range: 4–86 years) and a male-to-female ratio of approximately 1:1 (65 men and 63 women). Eighteen subjects of this cohort (14.1%) were classified as immunocompromised already before the time of imaging, due to an underlying condition and/or the use of immunosuppressive or immunomodulatory medication: 7 with human immunodeficiency virus infection, 4 with rheumatoid arthritis, 3 post-organ transplantation, 2 with multiple sclerosis, 1 with Crohn’s disease, and 1 with chronic lymphocytic leukemia (identified as Richter transformation). In our cohort, subjects with IP-LBCL (119/128, 93.0%) were more frequently observed (χ^2^ = 185.64, *P* < 0.001) than those with DLBCL with SCNSL (9/128, 7.0%). Significant age differences were found between both groups (primary: 71 years [59.5–76] vs. secondary: 58.2 years ± 9.0, W = 795.50, *P* = 0.015), while gender distribution was similar (χ^2^ = 0.00, *P* = 0.96). Although the odds of being immunocompromised were slightly higher in the IP-LBCL group compared to the DLBCL with SCNSL group (OR = 1.34, 95% CI: 0.16–63.20), this difference was not statistically significant (*P* = 1.00).

### Features of imaging data and clinical diagnosis

**Fig. 2:**
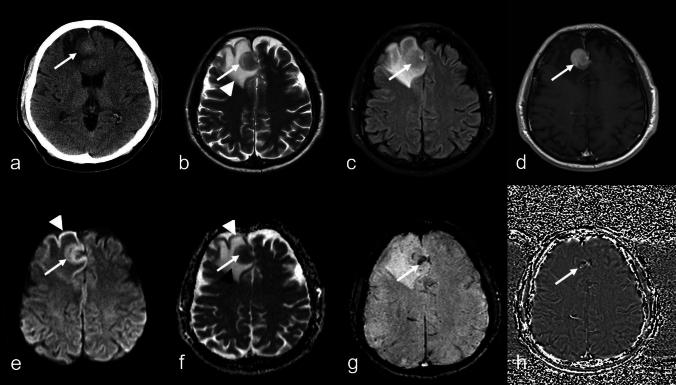
62-year-old man with primary large B-cell lymphoma of immune-privileged sites (IP-LBCL), negative for Epstein–Barr virus. **a:** Axial CT showing a slightly hyperdense intraaxial lesion (arrow); **b–h:** MRI showing an inhomogeneous hypointense cortical and subcortical solid lesion (**b, c:** T2-weighted imaging (WI), fluid-attenuated inversion recovery, arrows) with homogeneous contrast enhancement (**d:** T1WI; arrow) and restricted diffusion (**e:** diffusion-WI, b = 1000 s/mm^2^; **f:** apparent diffusion coefficient (ADC) map, mean ± SD: 0.69 ± 0.14 × 10⁻^3^ mm^2^/s, arrow; surrounding vasogenic edema (**f;** black arrowhead; ADC value mean ± SD: 2.2 ± 0.07 × 10⁻^3^ mm^2^/s; **g:** susceptibility-WI: confluent signal loss (arrow); **h:** phase image (arrow). Note the bandlike cortical diffusion restriction in the right frontal region (**e, f**; arrowheads), consistent with focal epileptic seizures

**Fig. 3:**
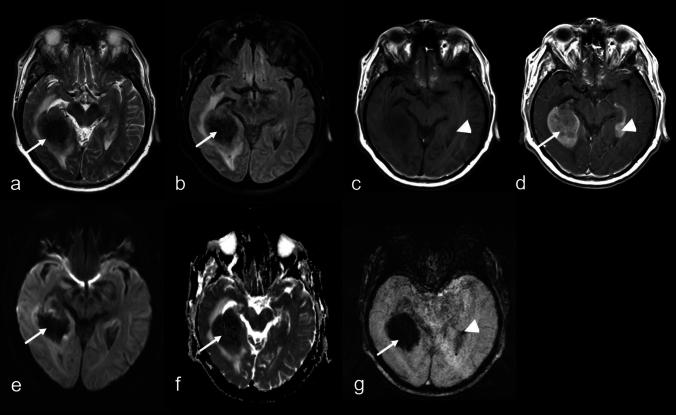
71-year-old man suffering from primary large B-cell lymphoma of immune-privileged sites (IP-LBCL), negative for Epstein-Barr virus. MRI disclosing a large solid temporooccipital right-sided lesion with conglomerate signal loss (**a**: T2-weighted imaging (WI); **b**: fluid-attenuated inversion recovery; **e, f**: diffusion-WI; **g**: susceptibility-WI; arrows.) Nearly homogeneous contrast enhancement (**c, d**: T1WI; arrows). Multifocal cerebral involvement is also noted, including a temporal lesion on the left side with punctate signal loss (**c, d, g**; arrowheads)

**Fig. 4:**
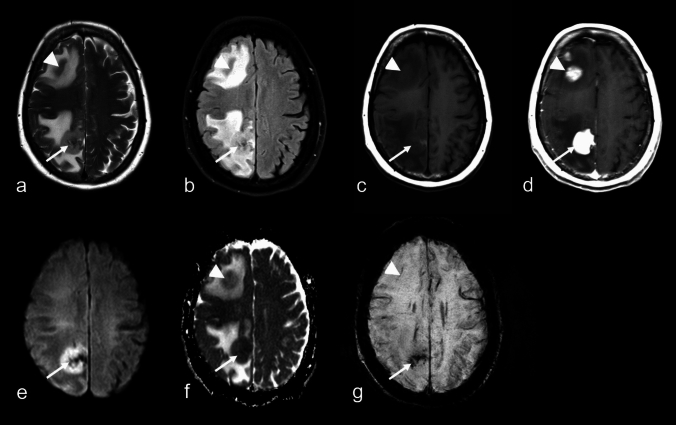
56-year-old woman with primary large B-cell lymphoma of immune-privileged sites (IP-LBCL), negative for Epstein-Barr virus. MRI (**a**: T2-weighted imaging (WI); **b**: fluid-attenuated inversion recovery; **c, d**: T1WI; **e, f**: diffusion-WI, apparent diffusion coefficient (ADC) map) reveal two lesions, one in the right frontal lobe (arrowheads) and one in the right parietal lobe (arrows), with perifocal edema and nearly homogeneous contrast enhancement (**d**). Susceptibility-WI shows confluent signal loss in the parietal lesion (**g**; arrow), while the frontal lesion exhibits no definite tumor-associated signal loss (**g**; arrowhead: linear signal loss corresponds to a vein). Note the different diffusion alterations between the lesions: parietal ADC mean ± SD: 0.53 ± 0.07 × 10⁻^3^ mm^2^/s (**f**; arrow); frontal ADC mean ± SD: 0.86 ± 0.14 × 10⁻^3^ mm^2^/s (**f**; arrowhead)

**Fig. 5:**
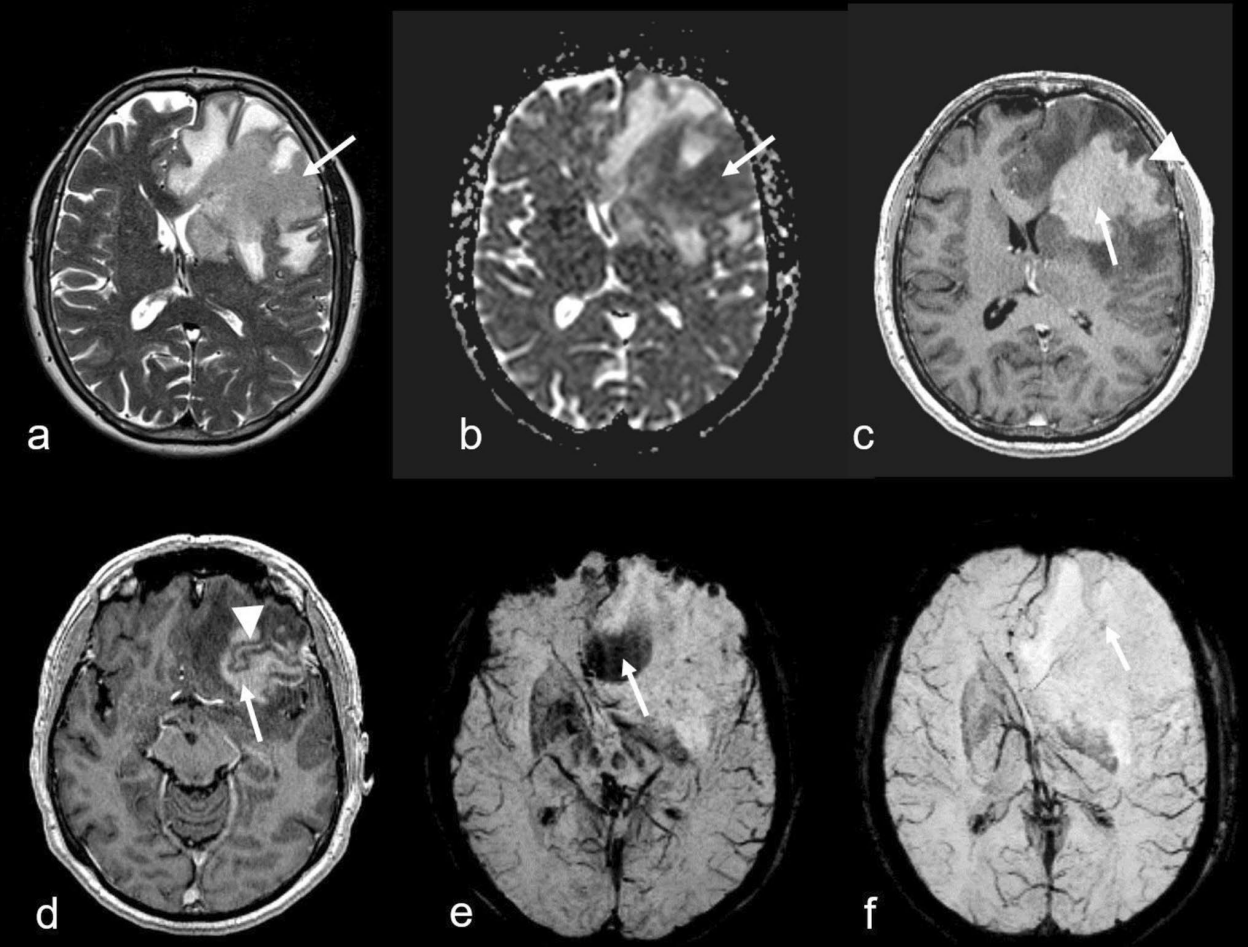
69-year-old woman presenting with progressive non-fluent aphasia caused by primary large B-cell lymphoma of immune-privileged sites (IP-LBCL), negative for Epstein–Barr virus. MRI reveals a large left-sided frontal lesion with associated midline shift (**a**: T2-weighted imaging (WI); arrow), inhomogeneous restricted diffusion (**b**: apparent diffusion coefficient map; mean ± SD: 0.75 ± 0.12 × 10⁻^3^ mm^2^/s; arrow), homogeneous contrast enhancement (**c, d**: T1WI; arrows) sparing the cortex (**c, d**; arrowheads), and focal areas of different signal loss patterns on susceptibility-WI (**e, f**; arrow)

Instructive cases illustrating the described imaging patterns are presented in Figs. 2, 3, 4, 5 and Supplementary Figures S1–S4. Of the 128 cerebral MRI datasets in our local Picture Archiving and Communication System that met the criteria, 59 (46.1%) were performed at our institute of neuroradiology, while 69 (53.9%) were imported from various external clinics and radiology practices. Of these sequence protocols analyzed, 90 (70.3%) included T2*WI, while 38 (29.7%) featured SWI. For cases with suspected IP-LBCL on MRI, the median time to intracranial biopsy was 8 days [5–13] (range, 0–69 days). None had received specific oncologic treatment beforehand. Among these 119 subjects, 88 (73.9%) had not received corticosteroids at the time of MRI, 22 (18.5%) had received an initial corticosteroid dose, and in 9 cases (7.6%), prior corticosteroid use was retrospectively unclear. The median time between a first biopsy-guided diagnosis of extracerebral DLBCL with SCNSL suspicion on MRI was 150 days [35–928] (range, 2–5259). In this group, five cases were confirmed post-imaging through biopsy of the intracranial manifestation or cerebrospinal fluid analysis (in cases of leptomeningeal spread) within a median of 8 days [7–11] (range, 6–71), while the remaining four cases were diagnosed solely based on imaging findings. Seven of nine SCNSL cases had completed prior treatment for extracerebral lymphoma, while one was under watch-and-wait several years after chemotherapy for chronic lymphatic leukemia, and another had not initiated therapy due to recent detection of neck lymphoma before brain manifestation.

### Analyses of imaging patterns

After reaching consensus, both raters identified 70 out of 128 cases (54.7%) with intratumoral SE on either T2*WI or SWI, achieving an almost perfect interrater agreement (κ = 0.89). SE were observed in 43 of 90 cases on T2*WI (47.8%) and in 27 of 38 cases on SWI (71.1%), indicating that SWI had significantly higher detection rates compared to T2*WI (χ^2^ = 4.94, *P* = 0.03). Among all subjects, 65 were positive and 54 negative for SE on T2*WI or SWI in the IP-LBCL group, while 5 were positive and 4 negative for the same findings in the DLBCL with SCNSL group. Thus, no significant association (*P* = 1.00) was observed between both groups and the observations on susceptibility-based imaging (OR = 0.96, 95% CI: 0.18–4.72). In the larger group of IP-LBCL, immunosuppression was the only significant predictor of susceptibility-related intratumoral changes (*P* = 0.001, OR = 16.10, 95% CI: 2.89–304.79, while neither age, gender, nor started corticosteroid treatment significantly influenced the odds. Notably, among the 18 immunosuppressed cases, only one patient, who had rheumatoid arthritis and was receiving corticosteroid and methotrexate treatment, showed no SE. The exclusion of the nine cases with 'unknown' corticosteroid status at the time of imaging did not significantly alter these results (for immunosuppression: *P* = 0.001, OR = 16.42, 95% CI: 2.93–311.96).

Out of 128 cases, 71 (55.5%) showed multifocal brain involvement, while all 9 cases of DLBCL with SCNSL were unifocal. There was no significant association between lesion focality and SE (χ^2^ = 0, *P* = 1). In unifocal lesions (57 cases, 44.5%), when SE were present, the distribution showed that confluent patterns were the most frequent (n = 15), followed by punctate (n = 12), conglomerate (n = 2), and ring-like (n = 1) patterns. Imaging morphologies on T2WI/ce-T1WI, including solid without necrosis (n = 31), solid with necrosis (n = 22), ring-like (n = 2), and cortical/pial (n = 2), did not show any significant association (*P* = 0.49) with specific susceptibility patterns, including cases with absent signal alterations. In 71 subjects with multifocal lesions, when SE were identified, the distribution showed punctate patterns were the most frequent (n = 20), followed by confluent (n = 14), ring-like (n = 3), conglomerate (n = 2), and linear (n = 2) patterns. In 25 subjects with multifocal lesions (35.2%), both present and absent SE were observed. Similar to unifocal lesions, the corresponding multifocal T2WI/ce-T1WI morphologies, including solid without necrosis (n = 19), solid with necrosis (n = 36), ring-like (n = 1), cortical/pial (n = 14), diffuse parenchymal enhancing (n = 9), diffuse perivascular enhancing (n = 11), diffuse subependymal enhancing (n = 4), and diffuse parenchymal with non- or scarcely enhancing patterns (n = 2), also did not show a significant association (*P* = 0.96) with different patterns of SE. In general, lesions with any necrotic components were observed in 61 subjects (47.7%), with a slight predisposition for cases with IP-LBCL (58/119) compared to DLBCL with SCNSL (3/9). However, this difference was not statistically significant (OR = 1.89, 95% CI: 0.38–12.24; *P* = 0.50). Notably, there was no significant association between necrosis and SE (χ^2^ = 0.58, *P* = 0.45).

## Discussion

This single-center, retrospective, observational study represents the largest cohort to date for analyzing susceptibility-based MRI sequences in a well-defined population, encompassing 128 patients with histologically confirmed DLBCL, predominantly with primary CNS manifestations, alongside fewer secondary cases. In this work, we provide compelling evidence that both reinforces and challenges previously accepted concepts about lymphomas. SE on MRI, once regarded as rare in IP-LBCL, are demonstrated to be more prevalent than traditionally thought. Our findings contribute significantly to the ongoing debate on the prevalence of SE, which were found to be substantial, observed in nearly half of cases on T2*WI and in almost three-quarters on SWI. Additionally, immunosuppressive status was confirmed as a key feature associated with SE within the tumors. Interestingly, already started corticosteroid therapy at the time of imaging showed no influence on the presence or absence SE, nor were these changes significantly associated with patient age or sex. Moreover, distinct morphologies on T2WI and ce-T1WI, including necrotic areas, demonstrated no clear relationship with SE. Multifocal findings further suggest that different patterns with or without these signal alterations may reflect various stages of lymphoma progression.

This study focused exclusively on DLBCL of the CNS (WHO ICD-10 code C83.3), the most common subtype of CNS lymphoma, to ensure a defined and clinically relevant diagnostic target. Other subtypes, such as intravascular large B-cell lymphoma, lymphoid granulomatosis, or Burkitt lymphoma and mantle-cell lymphoma with SCNSL, were excluded from the analysis. In our study cohort, IP-LBCL in immunocompetent patients was the most frequent presentation. DLBCL with SCNSL accounted for only 7% of cases in our study population, including one immunocompromised patient with Richter’s transformation of chronic lymphocytic leukemia. This marked imbalance is statistically significant but reflects real-world clinical patterns observed in our institutional experience. As primary and secondary CNS lymphomas are distinct disease entities, IP-LBCL being a primary CNS malignancy and SCNSL a rare manifestation of systemic DLBCL, direct comparisons are inherently limited. To our knowledge, no studies have provided a direct epidemiological comparison of their prevalence. Accordingly, comparisons between IP-LBCL and SCNSL in our study were conducted as a secondary analysis and should be interpreted with caution. We also noted a markedly higher proportion of multifocal cerebral manifestations (56%) compared to those reported in the literature (30–35%) [2], whereas all nine cases of DLBCL with SCNSL were unifocal. In line with current expert consensus, the diagnosis of secondary CNS involvement may be made based on systemic-site biopsy alone in case of synchronous systemic lymphoma and characteristic CNS findings on MRI, following expert neuroradiologic review [33]. In our cohort, 5 of 9 SCNSL cases had histopathological CNS confirmation, while 4 were diagnosed via this imaging-guided approach, introducing potential verification bias that should be considered when interpreting subgroup findings.

Image-guided suspicion of CNS lymphoma significantly impacts patient management. For instance, corticosteroid therapy in cases with symptomatic peritumoral edema should be avoided prior to biopsy whenever clinically feasible, as it can reduce the diagnostic yield of the histopathological finding [2, 4–7]. Among the participants in our analysis who had already received an initial dose of corticosteroid therapy, at least 22 versus 97 cases (with 9 cases having an unknown status) showed no significant influence of corticosteroid use on the presence or absence of SE on T2*WI or SWI. Our analysis found that only immunosuppression was a significant predictor of SE, consistent with findings in the literature [2, 4, 30–32]. Other potential confounders, such as tumor burden or lesion location, were not included in the multivariate analysis and may limit the completeness of confounder control.

High-grade gliomas and lymphomas often exhibit similar appearances on structural MRI, complicating their differentiation, a challenge particularly relevant for initial clinical workflows. Although no control group of non-lymphomatous CNS tumors was included, limiting direct assessment of SE specificity, previous comparative studies often relied on small lymphoma cohorts, potentially underestimating SE prevalence. By focusing on a large, well-characterized lymphoma population, this study aimed to more accurately define the true prevalence and imaging patterns of SE across lymphoma subtypes and immune states. Moreover, only ce-T1WI and T2WI were used to assess structural MRI features, as our focus was on evaluating morphologic characteristics such as necrosis, which was central to the study's scope. Although DWI and PWI can provide valuable information, these sequences were not consistently available across all protocols, particularly in external datasets, and were therefore not included in our analysis. Advances in PWI have been proposed to improve differentiation between both entities, despite limitations like reliance on post-processing [34]. Techniques, such as dynamic susceptibility contrast PWI, can identify tumor regions with characteristic blood flow patterns and contrast leakage, aiding differential diagnosis [35]. However, these parameters have not been demonstrated to be predictive of overall survival or progression-free survival [11]. Dynamic contrast-enhanced PWI, long considered less reliable due to variability in arterial input functions, has recently shown even improved accuracy when using derived measures, especially for differentiating glioblastoma with low relative cerebral blood volume from lymphomas [36]. Our study did not include an analysis of PWI and its associations with findings on T2*WI or SWI, as it was unavailable in most external datasets.

2D T2*WI and high-resolution 3D SWI are MRI sequences that rely on detecting SE as a key feature. Unlike other advanced imaging methods, these techniques do not require post-processing, making them straightforward and efficient tools for identifying abnormalities such as hemorrhage, calcification, presence of iron-laden microglia or macrophages, or free radicals produced by macrophages. We did not perform histopathological correlation to differentiate the underlying causes of SE, nor did we systematically analyze phase-sensitive images from SWI, which can help differentiate paramagnetic substances (e.g., hemorrhage, iron-laden macrophages) from diamagnetic ones (particularly calcifications). However, phase-sensitive images were unavailable for most external MRI scans and were therefore excluded from analysis. Compared to other cohort studies, our SE detection rate of 71.1% on SWI (27 of 38 cases) surpasses the previously highest reported rate of 53% (10 of 19 cases) by Sakata et al. [37], and far exceeds the 0% (0 of 7 cases) reported by Kim et al. [24], which represents the lower end of the spectrum. These differences may reflect technical factors, including the use of submillimetric in-plane resolutions ranging from 0.9 × 0.9 mm down to 0.3 × 0.3 mm in our in-house protocols, which likely enhanced the detection of subtle, particulary punctate SE. In our larger T2*WI dataset, SE were detected in 47.8% of cases (43 of 90), using in-plane resolutions of 0.7 × 0.7 mm and 0.4 × 0.4 mm. These findings place our study at the upper end of the reported spectrum.

In cases of CNS lymphomas, SWI has been proposed as valuable tools for distinguishing them from high-grade gliomas. In immunocompetent patients, the presence of hemorrhage on pretherapeutic imaging was considered a strong argument against a diagnosis of lymphoma [18–28]. This was supported by a 2013 study in which two radiologists did not identify any intratumoral SE in 14 cases of DLBCL, while one rare case of T-cell lymphoma, an entity with limited concrete imaging data, interestingly demonstrated a multifocal presentation with varying degrees of SE [28]. However, there were case reports describing primary lymphomas of the CNS presenting with intracerebral hemorrhages also in immunocompetent patients [38, 39]. Moreover, small hemorrhages were identified in 4 out of 10 immunocompetent patients with primary DLBCL of the CNS in one study [40], while intratumoral SE on SWI were observed in 6 out of 19 cases (32%) in another study [23]. The degree of susceptibility, evaluated using the grading scale proposed by Park et al. [27], revealed that only low-grade signals on SWI could reliably differentiate lymphomas from glioblastoma multiforme. Ozturk et al. showed that combining apparent diffusion coefficients and SWI signal intensity analyses can differentiate glioblastoma without necrosis from lymphomas and their genomic subtypes, with susceptibility signals detected in 14 of 31 (45.2%) primary CNS lymphoma cases [26]. In our study, which includes the largest cohort to date of 128 cases of DLBCL with primary and secondary cerebral manifestations in both immunocompetent and immunocompromised patients, we observed intratumoral signal loss in 47.8% of cases on T2*WI and in 71.1% of cases on SWI. We did not apply the grading system for intratumoral susceptibility signals or combined relative measurements on SWI, as we observed a broad spectrum of susceptibility patterns that extended beyond this classification (e.g., conglomerate or ring-like), and the relative measurements are not readily applicable to routine clinical practice.

Indeed, several methodological aspects of our study likely explain the higher detection rates of SE compared to earlier studies reporting their rarity. First, our analysis focused exclusively on a well-defined histological entity, DLBCL of the CNS, allowing for precise diagnostic inclusion. Second, susceptibility imaging was the sole imaging target, with systematic evaluation conducted by two experienced neuroradiologists specializing in neuro-oncologic MRI, using a consensus-based approach. Importantly, we defined a broader range of SE patterns beyond typical microhemorrhages, including punctate, linear, confluent, conglomerate, and ring-like appearances, thus capturing a wider imaging spectrum. Additionally, nearly one-third of patients were imaged with high-resolution 3D SWI, and many in-house protocols used submillimetric in-plane resolution (down to 0.3 × 0.3 mm), which likely enhanced sensitivity for subtle susceptibility-related changes. Finally, our cohort included both immunocompetent and immunocompromised individuals with primary and secondary CNS lymphoma, better reflecting real-world heterogeneity.

In conclusion, these findings reinforce that SE are a common imaging feature in CNS lymphoma and that their presence does not exclude the diagnosis. SE should be interpreted in the context of other established imaging characteristics to support accurate differential diagnosis and avoid premature exclusion of lymphoma in cases with atypical imaging findings. Moreover, the confirmation that immunosuppression, but not corticosteroid use, is significantly associated with SE is clinically relevant for biopsy planning.

This study has several limitations, with one key challenge being the heterogeneity of imaging datasets. This variability stems from the referral-based nature of our university center for tumor diseases over more than one decade. MR imaging was performed on three different internal scanners with varying field strengths and at multiple external clinics and radiology practices, resulting in differences in protocols and quality that may have impacted the consistency of the findings. A drawback is the reliance on subjective image analysis. However, this was mitigated by having two experienced neuroradiologists specializing in tumor imaging independently review the SE, with discrepancies resolved through a consensus-building process. Another limitation is the lack of a detailed correlation between biopsy results and imaging findings. While SWI phase imaging offers a qualitative approach to differentiating susceptibility sources, it was not consistently available across datasets. Furthermore, quantitative susceptibility mapping, which provides objective, voxel-wise magnetic susceptibility values, was not implemented in this study. This limits our ability to precisely characterize the nature of SE. Additionally, the analysis did not include a within-subject comparison of SWI and T2*WI, limiting the ability to directly compare their sensitivity for SE detection. The heterogeneity of the patient population should also be acknowledged. However, at the time of initial MRI, it is often unclear whether CNS involvement represents primary or secondary lymphoma, the latter of which may present synchronously with systemic disease. Moreover, both forms of CNS lymphoma can occur in immunocompetent as well as immunocompromised individuals. This real-world variability underscores the diagnostic complexity encountered early in the clinical work-up. Our study population consisted predominantly of immunocompetent patients with primary CNS manifestations, introducing potential bias due to the small sample sizes for DLBCL with secondary CNS manifestations and for patients with relatively rare underlying conditions in our region, such as acquired immunodeficiency syndrome. In this context, no explicit analysis was conducted on the cohort for Epstein-Barr virus histopathological or serological findings, which further limits the scope of interpretation of our prevalent findings of signal alteration. Diabetes, as another acquired, potentially chronic immunocompromising factor, was not further evaluated in this study. This and other potential limitations arise from the study's primary design not being prospective and the inability to conclusively investigate these aspects retrospectively. Multiple statistical tests were performed to explore imaging and clinical associations, without formal correction for multiple comparisons; therefore, results from subgroup and univariate analyses should be interpreted as exploratory and hypothesis-generating. Lastly, the lack of subanalysis for other imaging features, such as on DWI or PWI, represent another limitation.

## Supplementary Information

Below is the link to the electronic supplementary material.

67-year-old man with impaired fine motor function of the right hand caused by immunodeficiency-associated CNS lymphoma, positive for Epstein-Barr virus. The patient underwent orthotopic liver transplantation 22 years prior. A frontodorsal and precentral pial and cortical lesion with severe perifocal edema is noted (a, b: T2-weighted imaging (WI), fluid-attenuated inversion recovery; arrows), disclosing linear contrast enhancement (c, d: T1WI pre-, post-contrast; arrows), cortical diffusion restriction (e, f: diffusion-WI, b=1000 s/mm², apparent diffusion coefficient map; mean ± SD: 0.4 ± 0.05 × 10⁻³ mm²/s; arrows) and small linear signal loss on T2*WI (g; arrow) (JPG 181 KB)

Nearly 5-year-old child with progressive headache, nausea, and dizziness caused by perivascular spreading of a primary large B-cell lymphoma of immune-privileged sites (IP-LBCL), negative for Epstein-Barr virus. T2-weighted imaging (WI) (a) and fluid-attenuated inversion recovery images (b) demonstrate nearly symmetric hyperintense signal changes in the basal ganglia and thalamus (arrows), accompanied by focal linear signal loss on susceptibility-WI (c: arrow) along with vein-associated signal loss. Post-contrast T1WI (d–f) reveals distinct perivascular contrast enhancement (arrows) (JPG 189 KB)

45-year-old man presenting with his first secondary generalized epileptic seizure, with a known history of human immunodeficiency virus infection. Diagnosis: immunodeficiency-associated CNS lymphoma, positive for Epstein-Barr virus. Axial T2-weighted imaging (WI) (a), fluid-attenuated inversion recovery images (b), and T1WI (c, d) reveal a right frontal intraaxial solid lesion (arrows) with inhomogeneous hyperintense rim (arrowheads) without contrast enhancement (d; arrowhead) and central lowered signal intensity; e–g: susceptibility-WI demonstrates partially confluent linear (e; arrow; g: phase image; arrow) and confluent punctuate (f; arrow) signal loss (JPG 264 KB)

44-year-old woman presenting with progressive flaccid paraparesis and dizziness caused by immunodeficiency-associated CNS lymphoma, positive for Epstein-Barr virus, with a known history of human immunodeficiency virus infection. Axial T2-weighted imaging (WI) (a), fluid-attenuated inversion recovery images (b) and T1WI (c, d) demonstrate a frontal paramedian right-sided lesion and a frontodorsal paramedian left-sided lesion, each with a hypointense margin (a, b; arrows) and ring-like enhancement (c, d; arrows). Susceptibility-WI shows ring-like lowered signal intensity (e; arrow), and diffusion-WI (DWI) reveals distinct diffusion restriction (f, g: DWI, b=1000 s/mm²; apparent diffusion coefficient (ADC) map). The left-sided lesion has a mean ADC value (±SD) of 0.53 ± 0.07 × 10⁻³ mm²/s, while the right-sided lesion shows a mean ADC value (±SD) of 0.68 ± 0.09 × 10⁻³ mm²/s (JPG 199 KB)

Representative examples of the five susceptibility effect (SE) patterns identified on susceptibility-based MR imaging. SE were visually categorized into five distinct types: (a) punctate – small, isolated, dot-like regions (arrow; susceptibility-weighted image (SWI), 1.5T); (b) linear – small, isolated, line-like regions (arrow; T2*-weighted image (WI), 1.5T); (c) confluent – merged punctate and/or linear areas forming continuous regions (arrow; SWI, 3T); (d) conglomerate – dense clusters of SE (arrow; T2*WI, 1.5T); (e) ring-like – circular or partially circular signal loss surrounding a central area (arrow; T2*WI, 1.5T) (JPG 151 KB)

## Data Availability

No datasets were generated or analysed during the current study.

## Abbreviations

ce: Contrast-enhanced
CNS: Central nervous system
DLBCL: Diffuse large B-cell lymphoma
IP-LBCL: Primary large B-cell lymphoma of immune-privileged sites
PWI: Perfusion-weighted imaging
SE: Susceptibility effects
SCNSL: Secondary central nervous system lymphoma
SWI: Susceptibility-weighted imaging
WI: Weighted imaging
