# Supplementary figures and images for: Susceptibility- and T2*-weighted MRI features of CNS large B-cell lymphoma in a large single-center cohort: prevalence, patterns, and clinical associations
Source: J Neurooncol. 2025 Jul 12;175(1):231–41. doi: 10.1007/s11060-025-05124-8 (PMC12367949; doi:10.1007/s11060-025-05124-8)

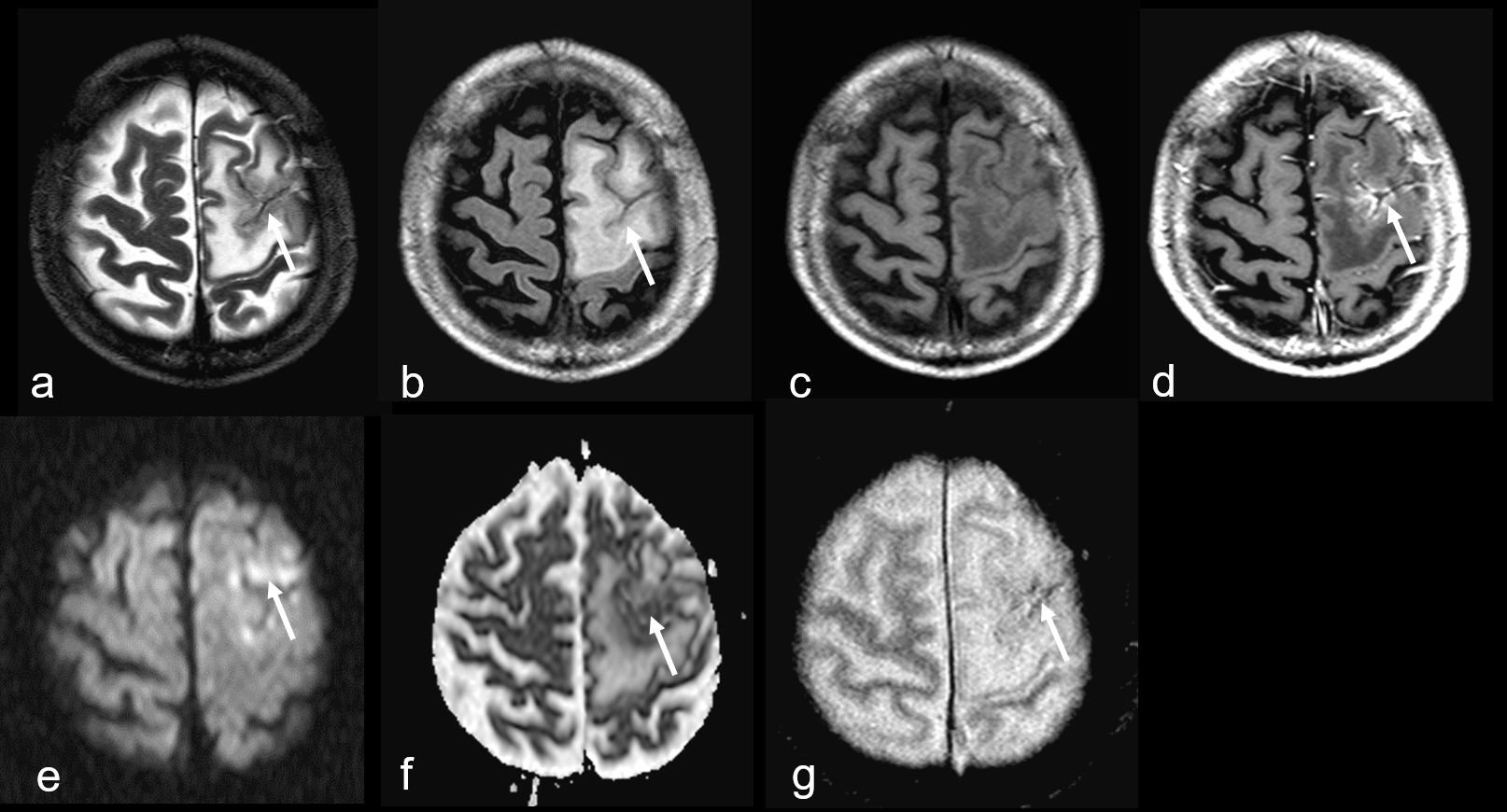

Supplement: Supplementary file 1 — 67-year-old man with impaired fine motor function of the right hand caused by immunodeficiency-associated CNS lymphoma, positive for Epstein-Barr virus. The patient underwent orthotopic liver transplantation 22 years prior. A frontodorsal and precentral pial and cortical lesion with severe perifocal edema is noted (a, b: T2-weighted imaging (WI), fluid-attenuated inversion recovery; arrows), disclosing linear contrast enhancement (c, d: T1WI pre-, post-contrast; arrows), cortical diffusion restriction (e, f: diffusion-WI, b=1000 s/mm², apparent diffusion coefficient map; mean ± SD: 0.4 ± 0.05 × 10⁻³ mm²/s; arrows) and small linear signal loss on T2*WI (g; arrow) (JPG 181 KB) [file 11060_2025_5124_MOESM1_ESM.jpg]

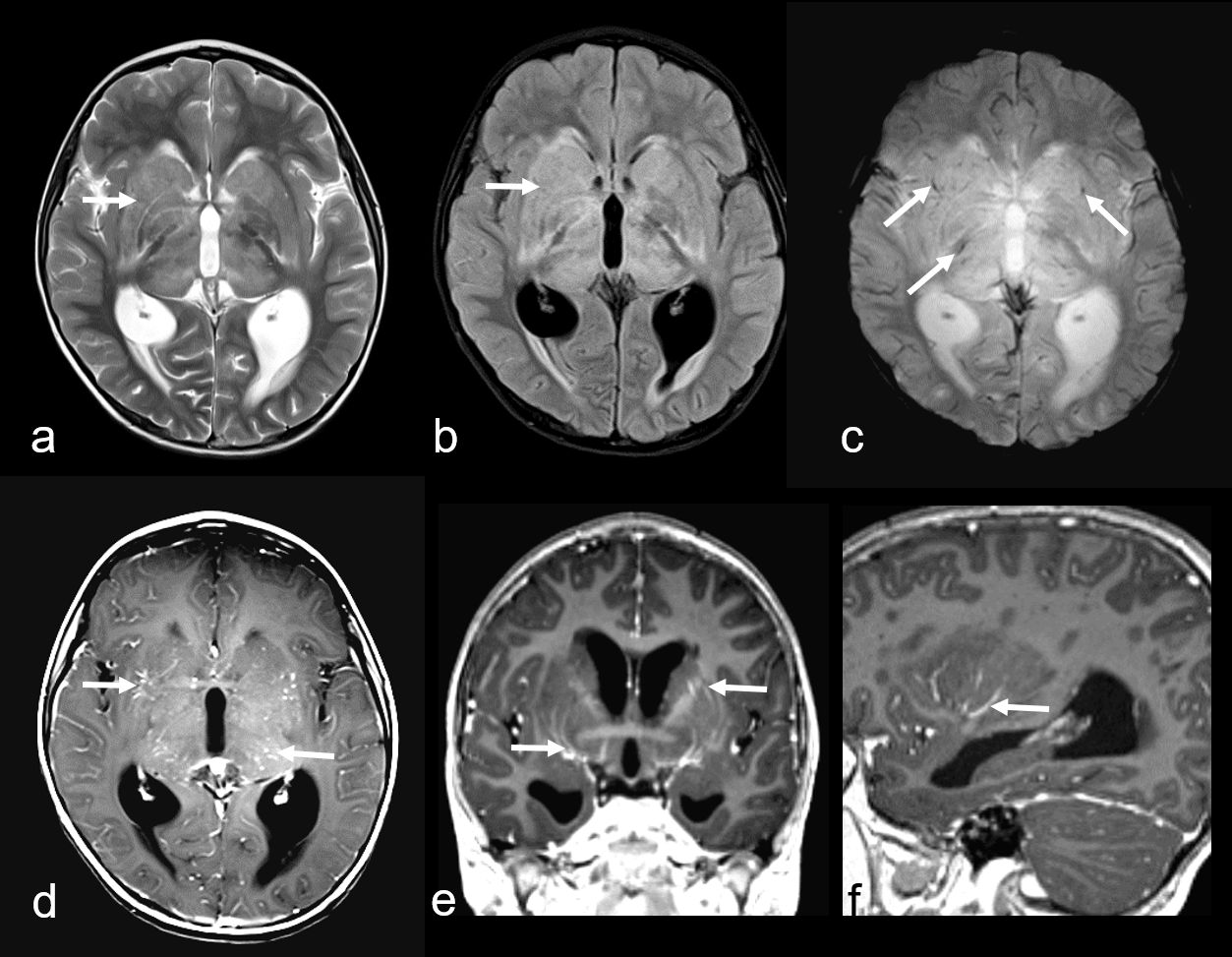

Supplement: Supplementary file 2 — Nearly 5-year-old child with progressive headache, nausea, and dizziness caused by perivascular spreading of a primary large B-cell lymphoma of immune-privileged sites (IP-LBCL), negative for Epstein-Barr virus. T2-weighted imaging (WI) (a) and fluid-attenuated inversion recovery images (b) demonstrate nearly symmetric hyperintense signal changes in the basal ganglia and thalamus (arrows), accompanied by focal linear signal loss on susceptibility-WI (c: arrow) along with vein-associated signal loss. Post-contrast T1WI (d–f) reveals distinct perivascular contrast enhancement (arrows) (JPG 189 KB) [file 11060_2025_5124_MOESM2_ESM.jpg]

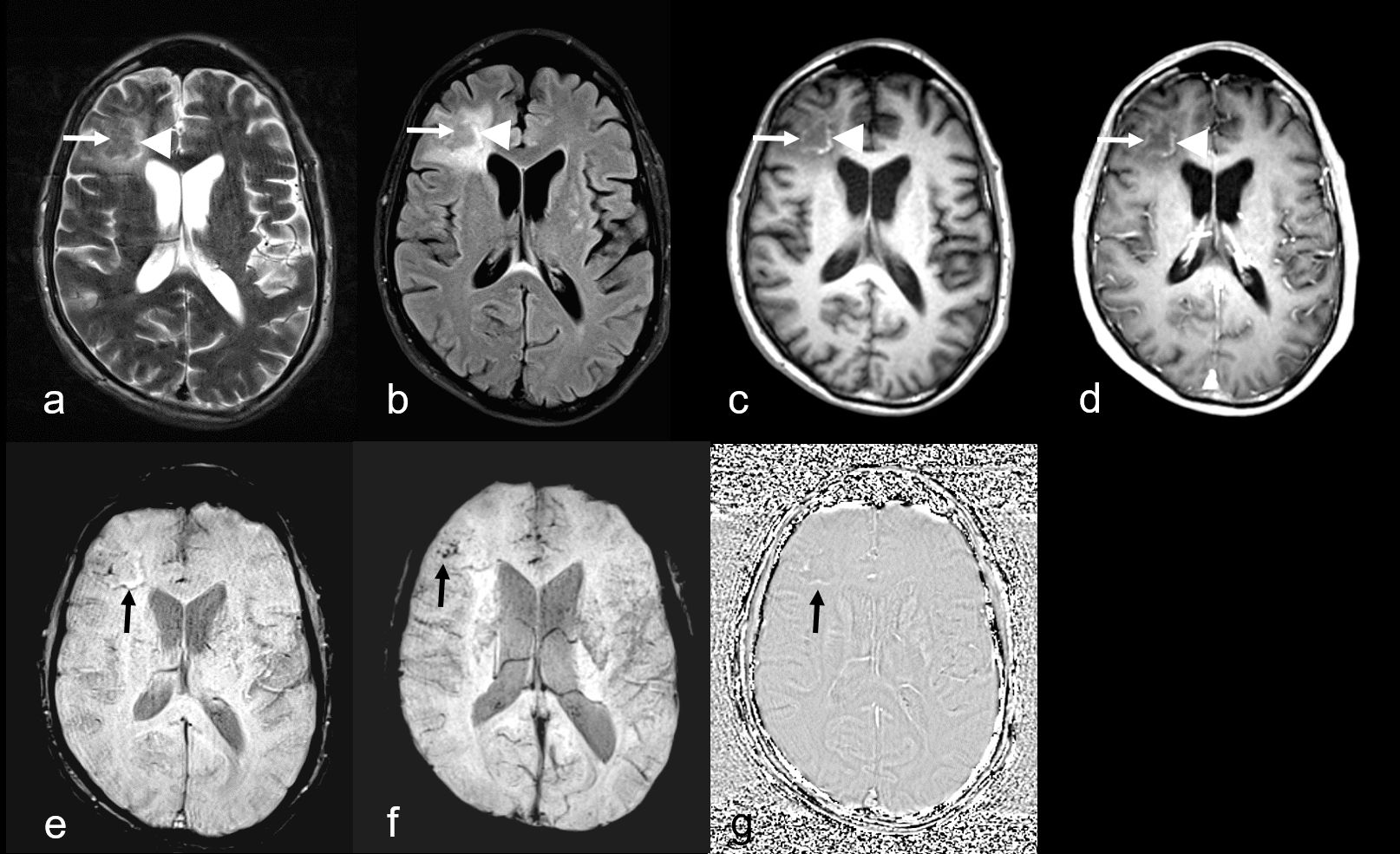

Supplement: Supplementary file 3 — 45-year-old man presenting with his first secondary generalized epileptic seizure, with a known history of human immunodeficiency virus infection. Diagnosis: immunodeficiency-associated CNS lymphoma, positive for Epstein-Barr virus. Axial T2-weighted imaging (WI) (a), fluid-attenuated inversion recovery images (b), and T1WI (c, d) reveal a right frontal intraaxial solid lesion (arrows) with inhomogeneous hyperintense rim (arrowheads) without contrast enhancement (d; arrowhead) and central lowered signal intensity; e–g: susceptibility-WI demonstrates partially confluent linear (e; arrow; g: phase image; arrow) and confluent punctuate (f; arrow) signal loss (JPG 264 KB) [file 11060_2025_5124_MOESM3_ESM.jpg]

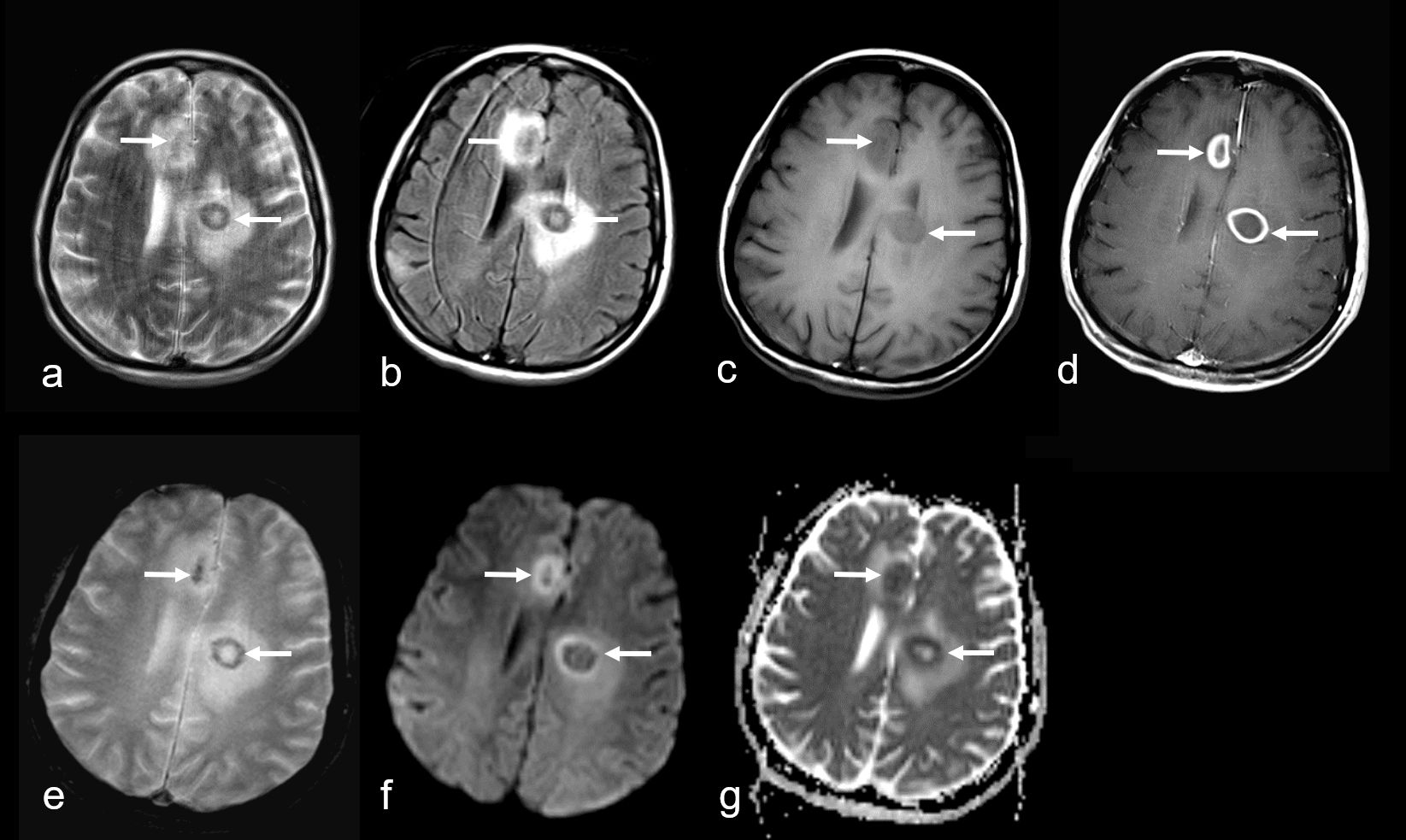

Supplement: Supplementary file 4 — 44-year-old woman presenting with progressive flaccid paraparesis and dizziness caused by immunodeficiency-associated CNS lymphoma, positive for Epstein-Barr virus, with a known history of human immunodeficiency virus infection. Axial T2-weighted imaging (WI) (a), fluid-attenuated inversion recovery images (b) and T1WI (c, d) demonstrate a frontal paramedian right-sided lesion and a frontodorsal paramedian left-sided lesion, each with a hypointense margin (a, b; arrows) and ring-like enhancement (c, d; arrows). Susceptibility-WI shows ring-like lowered signal intensity (e; arrow), and diffusion-WI (DWI) reveals distinct diffusion restriction (f, g: DWI, b=1000 s/mm²; apparent diffusion coefficient (ADC) map). The left-sided lesion has a mean ADC value (±SD) of 0.53 ± 0.07 × 10⁻³ mm²/s, while the right-sided lesion shows a mean ADC value (±SD) of 0.68 ± 0.09 × 10⁻³ mm²/s (JPG 199 KB) [file 11060_2025_5124_MOESM4_ESM.jpg]

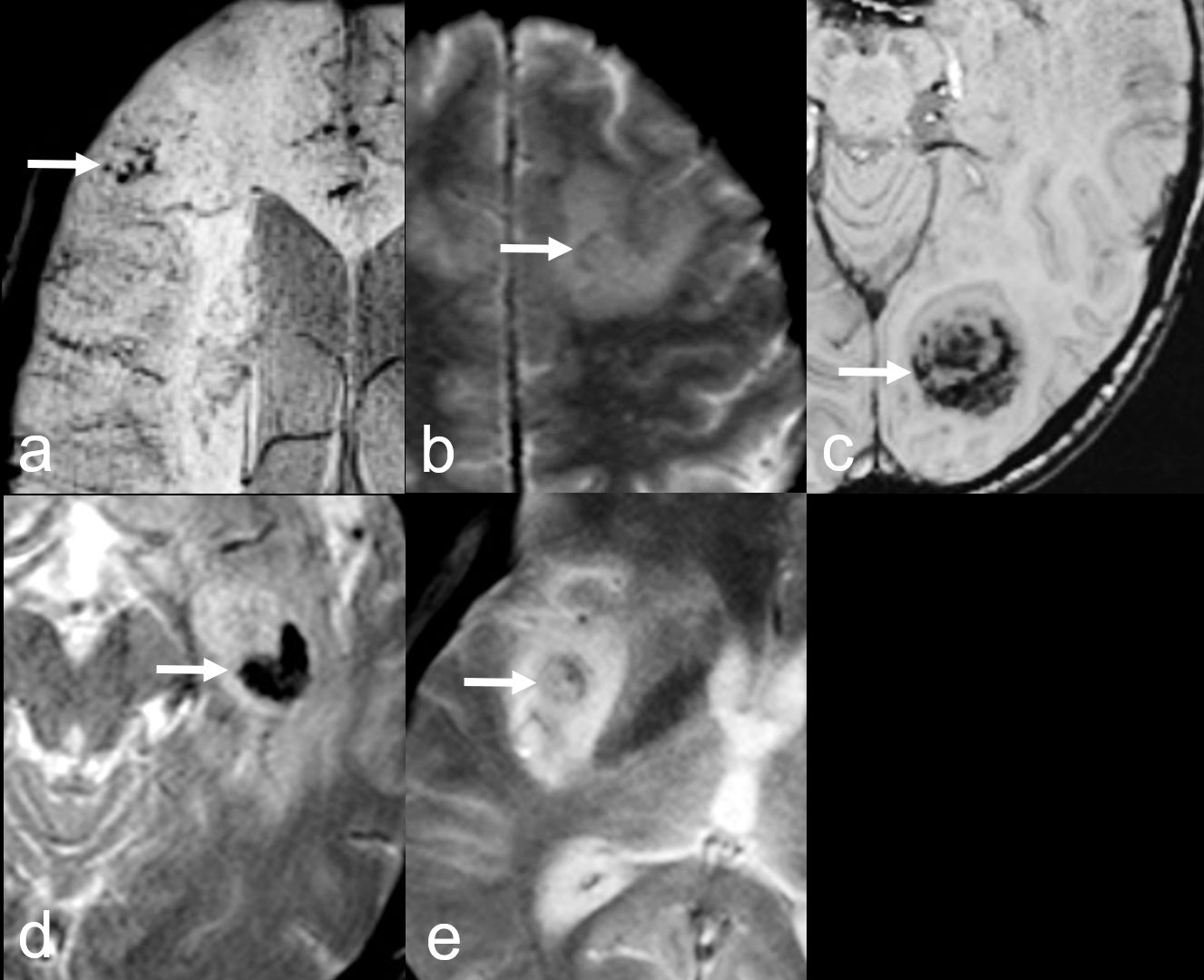

Supplement: Supplementary file 5 — Representative examples of the five susceptibility effect (SE) patterns identified on susceptibility-based MR imaging. SE were visually categorized into five distinct types: (a) punctate – small, isolated, dot-like regions (arrow; susceptibility-weighted image (SWI), 1.5T); (b) linear – small, isolated, line-like regions (arrow; T2*-weighted image (WI), 1.5T); (c) confluent – merged punctate and/or linear areas forming continuous regions (arrow; SWI, 3T); (d) conglomerate – dense clusters of SE (arrow; T2*WI, 1.5T); (e) ring-like – circular or partially circular signal loss surrounding a central area (arrow; T2*WI, 1.5T) (JPG 151 KB) [file 11060_2025_5124_MOESM5_ESM.jpg]
